# GP96 Interacts with HHV-6 during Viral Entry and Directs It for Cellular Degradation

**DOI:** 10.1371/journal.pone.0113962

**Published:** 2014-12-03

**Authors:** Bhupesh K. Prusty, Christine Siegl, Nitish Gulve, Yasuko Mori, Thomas Rudel

**Affiliations:** 1 Biocenter, Chair of Microbiology, University of Würzburg, 97074 Würzburg, Germany; 2 Graduate School of Medicine, Kobe University, Kobe 650-0017, Japan; German Primate Center, Germany

## Abstract

CD46 and CD134 mediate attachment of Human Herpesvirus 6A (HHV-6A) and HHV-6B to host cell, respectively. But many cell types interfere with viral infection through rapid degradation of viral DNA. Hence, not all cells expressing these receptors are permissive to HHV-6 DNA replication and production of infective virions suggesting the involvement of additional factors that influence HHV-6 propagation. Here, we used a proteomics approach to identify other host cell proteins necessary for HHV-6 binding and entry. We found host cell chaperone protein GP96 to interact with HHV-6A and HHV-6B and to interfere with virus propagation within the host cell. In human peripheral blood mononuclear cells (PBMCs), GP96 is transported to the cell surface upon infection with HHV-6 and interacts with HHV-6A and -6B through its C-terminal end. Suppression of GP96 expression decreased initial viral binding but increased viral DNA replication. Transient expression of human GP96 allowed HHV-6 entry into CHO-K1 cells even in the absence of CD46. Thus, our results suggest an important role for GP96 during HHV-6 infection, which possibly supports the cellular degradation of the virus.

## Introduction

Human Herpesvirus 6 (HHV-6) efficiently infects CD4^+^ T-lymphocyte and many other cell types *in vitro*. The two HHV-6A and -6B subtypes are highly similar in structure and infection mechanisms. However, they exhibit different tissue specificity. Cell surface receptor CD46 [1] and CD134 [2] serve as essential entry receptors for HHV-6A and HHV-6B, respectively, facilitating viral binding to human cells through direct interaction with different viral glycoproteins [3], [4]. Since CD46 and CD134 are expressed in nearly all-human cells, the engagement of this receptor cannot explain the host cell tropism of HHV-6. In addition, HHV-6 also infects cells in the absence of these receptors. Moreover, most of the cell types efficiently clear up the viral infection possibly by degradation of viral DNA. This led to the suggestion that these receptors might form a part of a larger multi-receptor complex and viral entry and viral DNA propagation and replication might depend on additional proteins [5].

Several arms of the innate immune defense of host cells are effective in preventing bacterial and/or viral replication (reviewed in [6]). Toll like receptors (TLRs) recognize components of pathogens and then respond by triggering an immune response [7]. HHV-6 impairs intracellular signaling and immunity of host cells by directly interfering with TLRs signaling [8]. TLRs modulate innate immunity in addition by binding to other key cell surface proteins like CD46, CD91, GP96 [9]. The common role of some of these key proteins in directing innate immune responses suggests that this virus subverts innate immune signaling through engagement of different co-receptors that ultimately decide the fate of virus infection.

In this study, we followed an unbiased proteomics approach to identify possible host cell proteins that directly or indirectly interact with HHV-6 during viral binding and/or entry. Through this approach, we identified host cell chaperone protein GP96 (also named as endoplasmin, grp94, TRA-1), a member of the Hsp90 family of proteins, as a key molecule for HHV-6 binding and entry, which also determines the subsequent viral DNA propagation. GP96 is mostly localized within the endoplasmic reticulum (ER) but has been observed on the cell surface in many different cell types [10], [11]. In addition to its role in the initiation of innate and adoptive immunity, GP96 also serves as receptor for vesicular stomatitis virus [12], *Listeria monocytogenes* [13], [14] and *Neisseria gonorrhoeae* [15]. We show here that viral infection increases cell surface exposure of GP96 that takes HHV-6 to a degradation pathway resulting in the loss of viral DNA.

## Materials and Methods

### Ethics statement

3 blood samples used in this study were provided by HHV-6 Foundation, USA collected with written informed consent under IRB# CI001-HHV6 approved by The Essex Institutional Review Board Committee, USA and by the Ethikkommission of the University of Würzburg, Germany.

### Cell culture

HeLa, HSB-2, Molt3 [16], [17] and HeLa.shGP96 [15] cells were maintained in RPMI 1640+5% fetal bovine serum (FBS) at 37°C and 5% CO_2_. CHO-K1 cells [15], [18] were maintained in F12 media with 10% fetal bovine serum.

### Virus stock preparation and infection

HHV-6A strain U1102 has previously been described [19]. HHV-6B strain Z29 was kindly provided by HHV-6 Foundation, USA. HHV-6A and HHV-6B were infected and propagated in HSB-2 and Molt3 cells, respectively, without antibiotics. Purified virus stocks were prepared by infecting the above mentioned suspension cells by the method described by Lu et al. [20]. Briefly, HHV-6 infected cells were mixed with uninfected HSB-2 or Molt3 cells at a ratio of 1∶10. When more than 80% of cells showed cytopathic effects visible under a light microscope, cell-free culture medium was harvested and filtered through a 0.45-µm filter and the virus was pelleted by centrifugation at 25,000×g for 3 h at 4°C. The virus pellet (called as semi-purified viral preparation hereafter) was suspended in cold Iscove's Modified Dulbecco's Media (IMDM) and frozen at −80°C until further use. Presence of intact viral particles was verified by electron microscopy of negative stained specimens. The HHV-6 titer expressed as the 50% tissue culture infective dose (TCID_50_) was determined by scoring the number of HSB-2 or Molt3 cells exhibiting cytopathic effects. The virus stock used had a titer of 10^6^ TCID_50_/ml. For viral infection experiments, cells were seeded for 24 h and then directly added with HHV-6 (10^6^ TCID_50_/ml for 10^6^ cells).

### PBMC isolation

Fresh PBMCs were first separated from whole blood using Histopaq1077 solution (Sigma-Aldrich, Germany) using manufacturer's instructions. Briefly, freshly collected blood was diluted 2 times with PBS and layered carefully onto 1 volume of Histopaq1077 solution in a falcon tube without mixing the solutions. PBMCs were collected after centrifugation at 600×g for 30 min at room temperature without applying brake. Cells were washed 3 times with 25 volumes of PBS and were kept in RPMI 1640 media with 5% fetal bovine serum (FBS) at 37°C and 5% CO_2_.

### HHV-6 binding assay

For the study of HHV-6 binding, host cells were added with HHV-6A in duplicates for 30 min on ice after which one set of virus added cells were washed extensively with PBS to remove unbound HHV-6A particles. The other set of cells were trypsinized and washed to remove the bound viral particles. Incubation of cells on ice allows viral binding and at the same time prevents viral entry, which was verified by using control cells that were trypsinized as mentioned above. Total DNA was extracted using DNAzol (Invitrogen) and subsequently processed for quantification of viral DNA amount by quantitative PCR (qPCR).

### HHV-6 viral entry assay

For the study of HHV-6 entry, host cells were added with HHV-6A in duplicates for 30 min on ice after which one set of cells were washed extensively with PBS to remove unbound HHV-6A particles and were transferred to a 37°C incubator with fresh media. The other set of cells were trypsinized and washed to remove the bound viral particles. These cells were subsequently transferred to a 37°C incubator with fresh media. Both the cell types were grown for another 2 h to permit viral entry. Incubation of cells on ice allows viral binding and at the same time prevents viral entry, which was verified by using control cells that were trypsinized as mentioned above. Transfer of cells to 37°C induces subsequent viral entry. Total DNA was extracted using DNAzol and subsequently processed for quantification of viral DNA amount by qPCR.

### Immunoblotting

HSB-2 cell lysate was prepared using RIPA buffer containing 50 mM Tris-HCl pH 7.5, 150 mM NaCl, 1% Triton X-100, 1% NP-40, 0.1% SDS, 10% Glycerol and protease inhibitor cocktail (Roche). Cell lysates were resolved by 8–12% sodium dodecyl sulfate (SDS)-poly- acrylamide gel electrophoresis. Proteins were transferred to polyvinylidene difluoride membranes (Millipore) and blocked with 10% skimmed milk. The membranes were then probed with respective primary antibodies and subsequently with HRP-conjugated secondary antibodies. Proteins were detected with peroxidase-coupled secondary antibody using the ECL system (Amersham). Antisera and antibodies used in this study are listed below.

### Quantitative PCR (qPCR)

For quantitative PCR (qPCR), PerfeCTa qPCR SuperMix (Quanta Biosciences) was used and PCR amplifications were done on a StepOnePlus real time PCR platform (Applied Biosciences) using manufacturer's protocol and SYBR Green chemistry. Amplified data were analyzed using StepOne Software v2.1. HHV-6 genome equivalents per cells was calculated by determining host cell number by amplification of the PI15 gene as described before [16].

### Cell Transfections

Transfections were performed using Lipofectamine 2000 (Invitrogen) and RNAiFect (Qiagen) following manufacturer's instructions. GP96 ON-TARGETplus smartpools siRNA were purchased from Dharmacon (Thermo Fisher Scientific, Europe). Mammalian expression vectors used for transient GP96 expression were described previously [15].

### Flow cytometry

Receptor expression during HHV-6 infection was analyzed using flow cytometry. After infection with *C. trachomatis* and/or HHV-6, adherent cells were detached using 5% EDTA in PBS. After fixation with 4% paraformaldehyde (PFA), nonspecific binding sites were blocked using 10% FCS in PBS. For primary antibody staining, cells were incubated with antibodies raised against human CD46 or GP96 for 1 h at 4°C. Cells were washed and subsequently stained with Cy2- or Cy5-conjugated secondary antibodies. After the final washing, cells were resuspended and analyzed with a BD Accuri C6 Flow Cytometer. Based on forward and side scatter characteristics (FSC/SSC), intact cells were detected and gated for further analysis. Fluorescence signals were detected in the FL-1 and FL-4 channel, relative fluorescence intensities (RFI) were quantified in histograms of FSC/SSC-gated cells (10,000 events). Appropriate isotype antibodies served as negative controls.

### HHV-6 immunoprecipitation

For HHV-6 immunoprecipitation experiments, HeLa protein lysates were prepared in PBS without any detergent. Cells were lysed in PBS containing protease inhibitors using glass beads. 10^6^ TCID_50_/ml of HHV-6 virus particles were incubated with 1 mg of protein lysate for 12 h at 4°C with constant gentle agitation. HHV-6 envelope glycoprotein antibodies were added to the mixture and incubated for another 2 h at 4°C. Pre-washed and blocked agarose beads (ROCHE) were added to the mixture and incubated for another 4 h at 4°C. Beads were washed 6 times with a wash buffer (20 mM Hepes, 200 mM NaCl, 1 mM EDTA containing 10 mg/ml BSA). In some cases, pre-clearing of protein lysates was carried out prior to incubation with HHV-6.

### Antibodies used

#### For Immunostaining and flow cytometry

Mouse monoclonal GP96 antibody raised against amino acids 676–803 of GP96 of human origin (sc-53929, Santa Cruz Biotechnology, USA), rabbit polyclonal GP96 antibody (1∶1000) raised against amino acids 200–411 of GP96 of human origin (sc-11402, Santa Cruz Biotechnology, USA), mouse monoclonal CD46 antibody (1∶200) raised against amino acids 35–328 of CD46 of human origin (sc-166159, Santa Cruz Biotechnology, USA).

#### For Western Blots and immunoprecipitation

Rabbit polyclonal GP96 (1∶1000) raised against amino acids 200–411 of GP96 of human origin (sc-11402, Santa Cruz Biotechnology, USA) and mouse monoclonal Actin antibody against human beta-actin were used for Western blot analysis. Immunoprecipitation was performed with mouse monoclonal gp60/110 (glycoprotein gB) antibody raised against HHV-6 (sc-57805, Santa Cruz Biotechnology, USA), Mouse monoclonal gp116/64/54 (glycoprotein gH) antibody raised against protein gp116/64/54 of strains -A and -B of HHV-6 origin (sc-65449, Santa Cruz Biotechnology, USA), mouse monoclonal HHV-6 gQ1 (gp82/105) antibody [21], rabbit monoclonal gM antibody [22] and rabbit monoclonal gB antibody [23]. Anti-HHV6 p41 MAb (9A5D12) was obtained from NIH AIDS repository. Rabbit polyclonal antibody against human Glucose 6-phosphate dehydrogenase (G6PD) (sc-67165) was obtained from Santa Cruz Biotechnology, USA.

#### For viral binding and competition assay

Mouse monoclonal GP96 antibody raised against amino acids 676–803 of GP96 of human origin (sc-53929, Santa Cruz Biotechnology, USA), rabbit polyclonal GP96 antibody raised against amino acids 200–411 of GP96 of human origin (sc-11402, Santa Cruz Biotechnology, USA), mouse monoclonal CD46 antibody raised against amino acids 35–328 of CD46 of human origin (sc-166159, Santa Cruz Biotechnology, USA).

### Immunostaining

For immunostaining, cells were fixed in 4% paraformaldehyde (PFA) for 30 min. Whenever required, cells were permeabilized with PBS +0.2% Triton X-100 for 20 min at RT. Cells were blocked with PBS +10% FBS for 1 h at RT and then stained with a primary antibody diluted in PBS +2% FBS at RT for 1 h. Cells were washed with PBS thrice and incubated with a Cy2/Cy3-labeled secondary antibodies and Draq5 in PBS +2% FBS for 1 h in the dark at RT. Slides were washed twice in PBS, once in distilled water to remove PBS and were mounted on Mowiol (Carl Roth). For confocal laser scanning microscopy, samples were analyzed using a Leica TCS SPE equipped with 488, 532, and 635 nm solid-state lasers for excitation. Images were taken using appropriate excitation and emission filters for the fluorescence dyes. Overlay images of the single channels were obtained using ImageJ.

### Statistical analysis

Statistical significance (p values) of acquired data was calculated using two-sided Student's t-test.

## Results

### GP96 interacts with HHV-6A and HHV-6B viral particles

**Figure 1:**
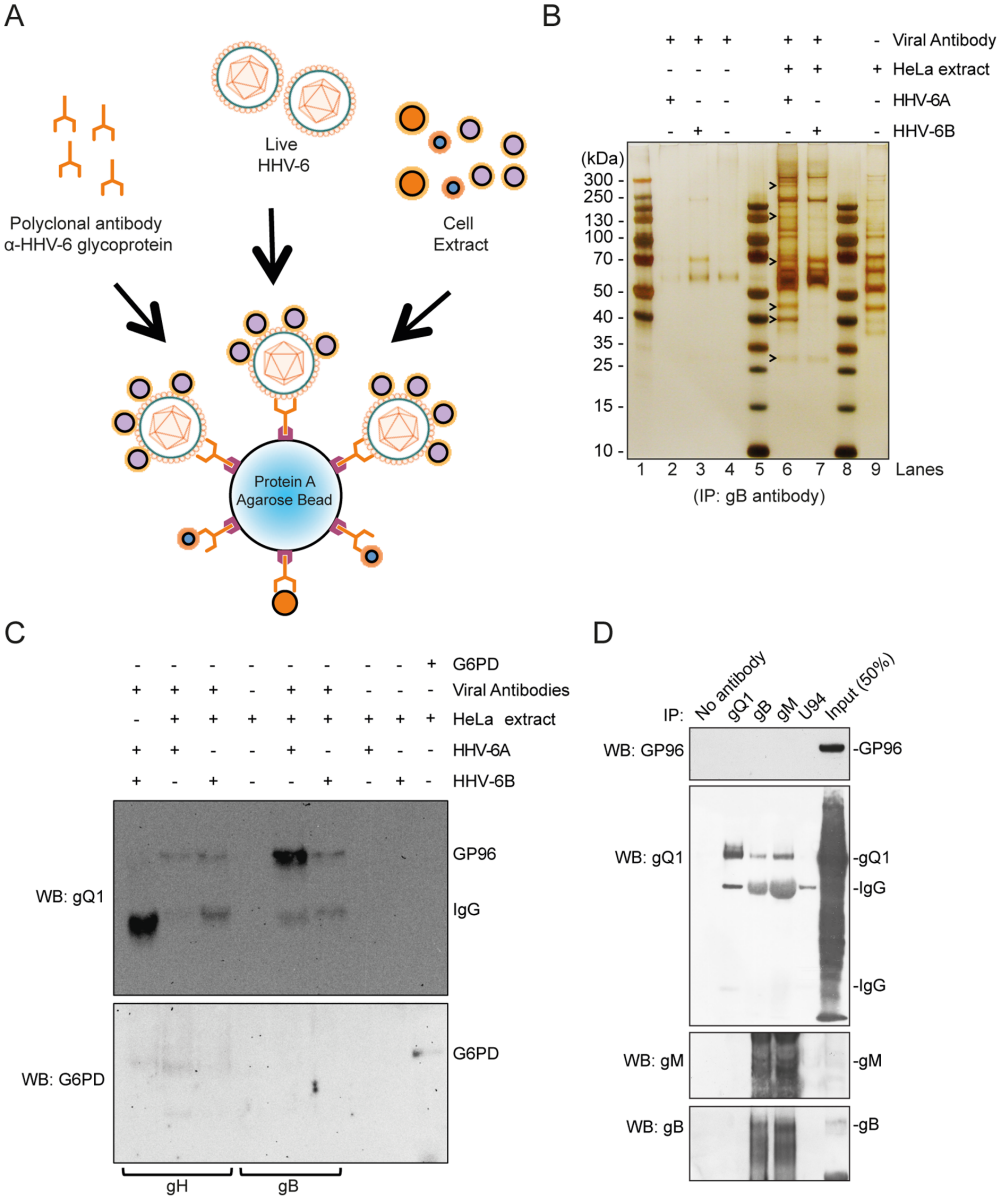
Identification of HHV-6-interacting host cell proteins. (**A**) Diagrammatic representation of immunoprecipitation (IP) experiment to detect unknown HHV-6-interacting host cell proteins. (**B**) Protein complexes directly interacting with HHV-6 viral particles were isolated by IP using anti-HHV-6 gB antibody, separated by SDS-PAGE and visualized by silver staining. Molecular weight markers are indicated on the left. Arrowheads point to some of the proteins differentially identified by mass spectrometry. Lane 1, 5 and 8 shows pre-stained protein ladders. (**C**) IP and Western blotting were carried out to validate and characterize the interaction between GP96 and HHV-6 envelope glycoproteins. As negative control no antibody (Ab), no HHV-6, no HeLa lysate were used for IP. An antibody against human Glucose-6-P dehydrogenase (G6PD) in the absence of viral particles was also used as a control. The Western blot of the IP samples was probed for human GP96 and subsequently with G6PD. Anti-HHV-6 gB and anti-HHV-6 gH antibodies were used for IP. (**D**) Lysates of HSB-2 cells infected with HHV-6A were used for IP, followed by Western blotting (WB) to detect interaction between GP96 and HHV-6 glycoprotein complexes. The blot was probed with GP96, gQ1, gM and gB antibodies. IgG heavy and light chains are marked at appropriate places.

To identify potential new host cell receptors for HHV-6, we followed an imunoprecipitation approach using semi-purified HHV-6 preparations (Figure 1A). Host cell proteins that interact with viral envelope were immunoprecipitated by using an antibody directed against viral glycoprotein gB (Figure 1A) and precipitated proteins were separated to select enriched protein bands (Figure 1B and Figure S1), which were later identified by mass spectrometry. This approach would also allow immunoprecipitation of host cell proteins that directly bind to viral glycoproteins present outside the intact viral particles. With this approach, we identified at least 11 different host cell proteins that were not co-immunoprecipitated with any of the control samples used (Table 1). Interestingly, we identified 2 putative receptor proteins, 4 proteins involved in host cell glycolytic pathways and 3 proteins of the host cell translation machinery. In this manuscript, we analyzed one of these proteins, GP96, for its involvement in viral binding, entry and survival.

**Table 1:** Host proteins differentially immunoprecipitated with both HHV-6A and HHV-6B.

Accession number	Protein Name	Possible Functions
IPI00942979.1	Transketolase	Enzyme of the Pentose phosphate pathway and Calvin cycle
IPI00219018.7	Glyceraldehyde-3-phosphate dehydrogenase	Enzyme involved in Glycolysis. Also helps in vesicular transport.
IPI00847989.3	Pyruvate kinase	Enzyme involved in Glycolysis.
IPI00465248.5	Isoform alpha-enolase of Alpha-enolase	Enzyme involved in Glycolysis. Also acts as a receptor on cell surface.
IPI00027230.3	Endoplasmin (GP96)	Helps in ER transport, also as a receptor on the cell surface.
IPI00455383.4	Clathrin heavy chain 1, Isoform 2	Forms specialized vesicles and pits on the inner surface of plasma membrane.
IPI00022881.1	Clathrin heavy chain 2, Isoform 1	Specialized function in endosomal sorting.
IPI00937615.2	Elongation factor 1-gamma	Translation machinery
IPI00186290.6	Elongation factor 2	Translation machinery
IPI00941522.1	Elongation factor 1-alpha	Translation machinery
IPI00894365.2	Highly similar to Actin, cytoplasmic 1	Cytoplasmic beta actin, involved in cell motility.

By using the above immunoprecipitation method, GP96 was co-immunoprecipitated with three different antibodies against viral glycoproteins (gQ1, gB and gH) (Figures 1B, 1C). To exclude an unspecific binding of GP96 to antibodies, a control immunoprecipitation experiment using an antibody against Glucose-6-phosphate dehydrogenase (G6PD) (Figure 1C) was performed in parallel. No GP96 precipitated in these experiments supporting a specific co-precipitation of GP96 with viral proteins. Since it has been shown that the viral glycoprotein complex gQ1, gQ2, gH and gL directly interacts with CD46 for HHV-6A entry [24], we tested whether the same glycoprotein complex also directly interacts with GP96. In addition, we also tested other viral glycoproteins like gB and gM for the possibility of direct interaction with GP96. For this we utilized total protein lysates from HHV-6A infected HSB-2 cells to co-immunoprecipitate GP96 but could not observe any detectable interaction of GP96 with these glycoproteins (Figure 1D). These results suggested that GP96 interacts with an unknown viral factor, which facilitates binding of host cell GP96 to HHV-6 viral particles.

### GP96 is exported to cell surface upon HHV-6 infection to allow viral attachment

**Figure 2:**
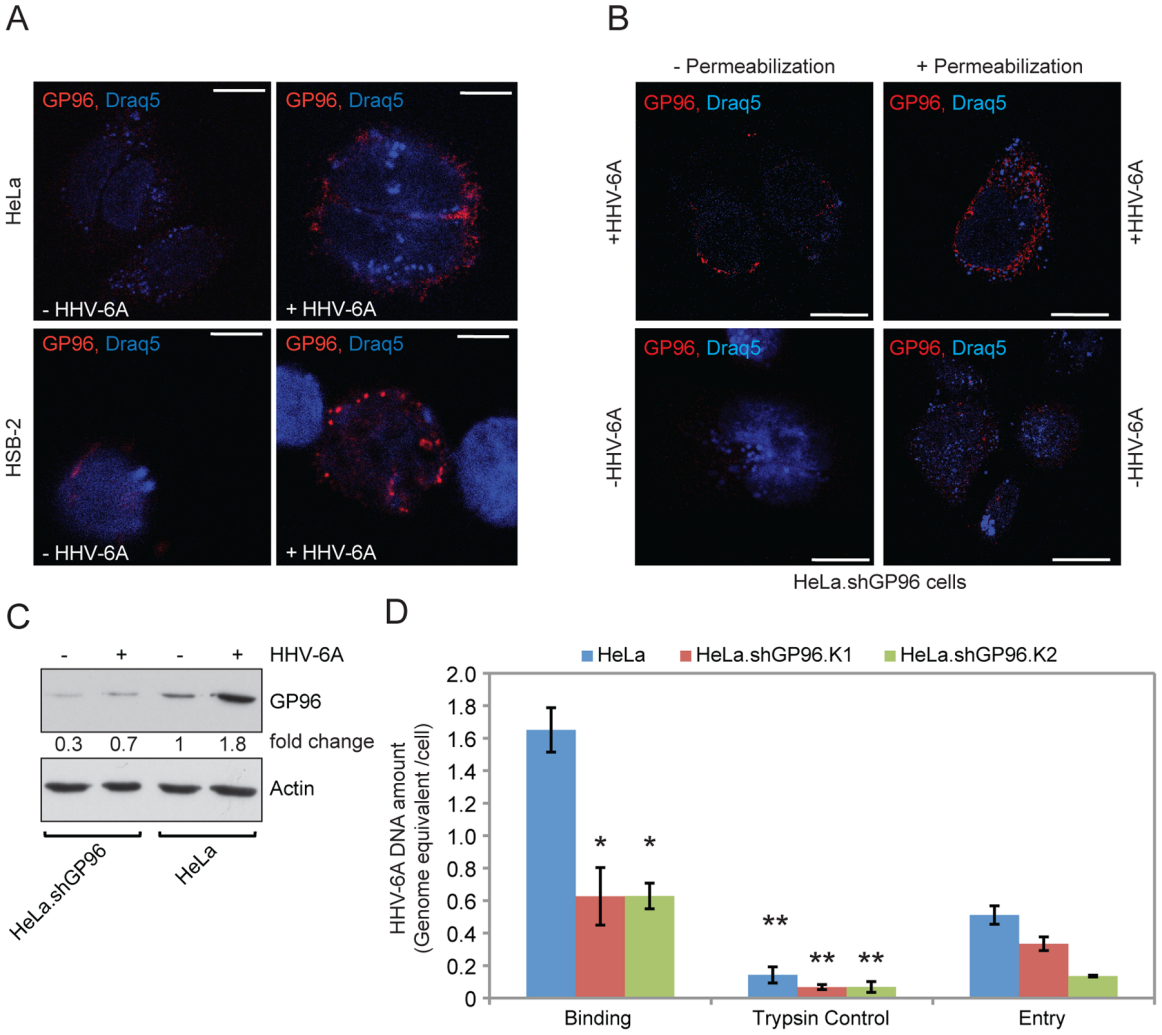
GP96 interacts with HHV-6. (**A**) Cell surface expression of GP96 increases in response to HHV-6 infection. HeLa and HSB-2 cells were infected with HHV-6A for one hour and analyzed by confocal laser microscopy. Draq5 staining was used to visualize nuclei. (**B**) GP96 expression is induced immediately after HHV-6A infection. HeLa cells carrying stable knock down of GP96 (HeLa.shGP96) were infected with HHV-6A for one hour. Non-infected cells served as control. Cell surface expression of GP96 was studied in non-permeabilized cells by immunofluorescence using a primary antibody against C-terminal end of GP96 and a Cy-3 conjugated secondary antibody. Total GP96 expression was studied in permeabilized cells. The scale bar represents 10 u˜ (**C**) Increase in total GP96 expression during HHV-6A infection. HeLa cells as well as HeLa.shGP96 cells were infected with HHV-6A for 48 h. Total GP96 expression was analyzed by Western blotting. Actin served as loading control. (**D**) Decrease in cell surface GP96 expression decreases HHV-6 binding and its subsequent entry. HeLa cells wildtype and GP96 knockdown cell lines (HeLa.shGP96 clone K1 and K2) were infected with HHV-6A to study viral binding and entry. Cells trypsinized to remove bound viral particles served as control. Cells were processed as described in Materials and Methods and viral DNA was quantified by qPCR. Data represent the mean ± SEM of three independent experiments. *, p≤0.05 and **, p≤0.005. Statistical analysis was based on the Student t-test.

Co-immunoprecipitation with HHV-6 glycoproteins suggested that GP96 might play a crucial role during HHV-6 binding and/or entry. As cell surface display of GP96 should then be an important requirement, we verified the surface expression of GP96 by confocal laser microscopy. HHV-6 has a non-replicative (non-lytic) life cycle in HeLa cells but efficiently replicates and forms viral particles in HSB-2 cells. Hence, we selected these two cell types for further analysis. GP96 was present at low amounts on the surface of HeLa and HSB-2 cells but its surface expression was rapidly increased in response to HHV-6 infection (Figure 2A). Interestingly, cells with a shRNA-mediated stable suppression of GP96 expression (HeLa.shGP96) and strongly decreased cell surface GP96 expression [15], also showed a transient increase in total as well as cell surface GP96 expression within 1 h of HHV-6 infection (Figure 2B). This might be due to a rapid increase of GP96 transcripts, which cannot be immediately down regulated by shRNA-mediated degradation. Increase in total expression of GP96 was confirmed by immunoblotting in HeLa, HeLa.shGP96 (Figure 2C) cells. Hence, our results indicate an increase in total expression as well as transport of GP96 to the cell surface upon HHV-6 infection.

### GP96 supports viral binding and entry

To further investigate the function of GP96 for HHV-6 binding, viral binding assays (see Materials and Methods) were performed in HeLa cells and in two different single HeLa cell clones with a stable knock down of GP96 (HeLa.shGP96). After 30 minutes incubation on ice together with HHV-6A viral particles, we observed up to 60% less HHV-6A binding to HeLa cells in the absence of GP96 (Figure 2D). Viral binding to the HeLa cell surface without a significant entry was verified by trypsinizing a parallel set of cells after 30 minutes incubation on ice (middle panel in Figure 2D), which removed up to 95% of the bound virus. GP96 knock down efficiency in all the cell types was determined by Western blotting (Figure S2). Similarly, viral entry was decreased in the absence of GP96 (Figure 2D), probably as a consequence of decreased viral binding. Thus our results suggest a possible role for GP96 in viral binding and subsequent entry.

### GP96 interferes with viral DNA maintenance and replication

**Figure 3:**
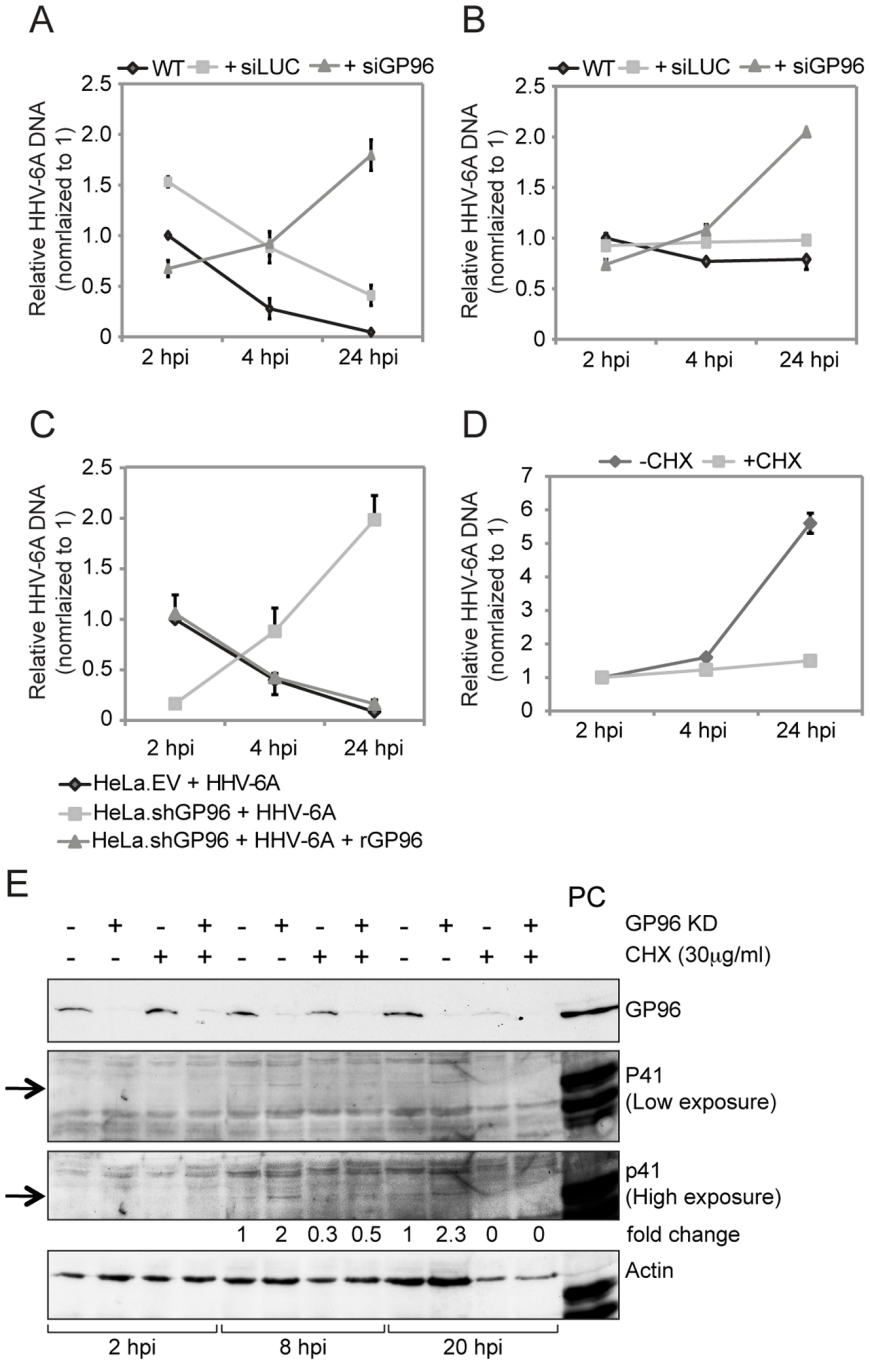
Absence of GP96 induces viral replication. (**A**) Decrease in cell surface GP96 expression decreases HHV-6 entry but increases subsequent replication of viral DNA. HeLa cells were transfected with siRNA against human GP96. In parallel, cells were transfected with siRNA against the luciferase gene (siLUC) as a control. Blank transfected as well as siGP96 and siLUC transfected cells were washed thoroughly and infected with HHV-6A for 2 hrs. After 2 hrs of viral infection, cells were washed with trypsin and extensive washing with PBS to remove any bound but non-entered HHV-6 particles. Cells were then incubated for different time intervals in the absence of viral particle in the culture media and subsequently processed for quantification of viral DNA amount by qPCR. (**B**) The experiment described under (A) was performed in HSB-2 cells. (**C**) HHV-6A entry decreases in absence of GP96. HeLa cells carrying lentivirus-mediated stable knock down of GP96 (HeLa.shGP96) and vector control (HeLa.EV) were infected with HHV-6A. In parallel, HeLa.shGP96 cells were transiently transfected with a construct expressing human GP96 (rGP96) for 24 h and were also infected with HHV-6A. Cells were processed as mentioned above and amount of viral DNA in these cells was measured by qPCR. (**D**) Increase in viral DNA replication in the absence of GP96 requires host cell translation. HeLa.shGP96 cells were infected with HHV-6A in presence or absence of cycloheximide (CHX) and subsequently analyzed by qPCR. Data represent the mean ± SEM of three independent experiments. **, p≤0.005. Statistical analysis was based on the Student t-test. (**E**) Viral gene expression is increased in the absence of GP96. HeLa and HeLa.shGP96 cells were infected with HHV-6A for different time intervals in the presence or absence of CHX. Western blotting was carried out to detect HHV-6 early antigen p41. Actin served as loading control. 41 kDa bands specific for HHV-6 p41 is marked with arrow. HHV-6A infected HSB-2 cell lysate was used as a positive control (PC).

The role of GP96 on the intracellular fate of HHV-6 was then investigated in HeLa (non-productive HHV-6 infection) as well as HSB-2 cells (productive HHV-6 infection). GP96 was silenced in these two cell types (Figure S2) and HHV-6A was added for 2 h to allow viral entry. Infected cells were trypsinized and washed thoroughly to remove extra cellular viral DNA. These cells were cultivated further in the absence of new viral infection and the amount of viral DNA was quantified by qPCR at different time points. In HeLa cells, the amount of viral DNA was reduced within 24 h of infection (Figure 3A), indicating a possible cellular defensive mechanism that destroys HHV-6A DNA inside the cell. As previously observed (Figure 2D), silencing of GP96 could not block viral entry completely but reduced amounts of viral DNA were detected inside HeLa cells at an early time point of 2 h (Figure 3A). Interestingly, the amount of viral DNA was increased up to three-fold within the first 24 h of viral infection indicating enhanced maintenance and possible replication of viral DNA in the absence of GP96. This effect was not due to transfection artifacts since the course of HHV-6 infection of control-transfected cells was similar to that of non-transfected cells (Figure 3A). Similarly, the amount of virus at early infection time points was similar or slightly lower in GP96-silenced HSB-2 cells in comparison to non- or control-transfected cells but increased up to 2-fold within 24 h of viral infection (Figure 3B) as observed in HeLa cells. But in contrast to HeLa cells, viral DNA was found to be stable for 24 h in HSB-2 cells under normal HHV-6A infection, supporting productive viral replication. These data suggested that GP96 possibly together with other proteins determines the fate of viral DNA by directing it to a degradation pathway.

To exclude off-target effects of GP96 silencing, GP96 was expressed in HeLa.shGP96 viral entry and maintenance was determined. Over-expression of recombinant GP96 in HeLa.shGP96 cells (Figure S2) resulted in increased viral entry (Figure 3C) thus supporting a role of GP96 in HHV-6 entry. In addition, recombinant GP96 expression in HeLa.shGP96 cells mimicked the viral infection as observed in HeLa cells (Figure 3C).

Epithelial cells like HeLa do not support productive viral infection. To investigate if the increase in viral DNA in the absence of GP96 was due to viral replication, infected cells were treated with cycloheximide (CHX), an inhibitor of host protein synthesis. In the presence of CHX, viral DNA did not increase in HeLa.shGP96 cells (Figure 3D), confirming increased viral replication mediated by host cell protein synthesis. We have previously shown that several viral genes are transcribed within 24 h of infection in HeLa cells [19]. Since efficient viral protein synthesis has not been demonstrated in HeLa cells before, production of HHV-6A early protein p41 was tested in both HeLa as well as HeLa.shGP96 cells [25], [26]. HHV-6 DNA binding protein p41 was detected within 8 h of viral infection in HeLa cells, which was up to 2-fold increased in HeLa.shGP96 cells (Figure 3E). This difference in p41 expression was further enhanced after 20 h of viral infection. CHX treatment prevented p41 expression in both cell types, confirming a role of host cell protein synthesis for viral DNA replication. In addition, we detected p41 in the nuclei of HeLa.shGP96 but not in nuclei of HeLa cells at 4 days of viral infection (Figure S2), further supporting our previous observation of increased viral replication in the absence of GP96.

### The C-terminus of GP96 directly interacts with HHV-6 and is necessary for viral entry

**Figure 4:**
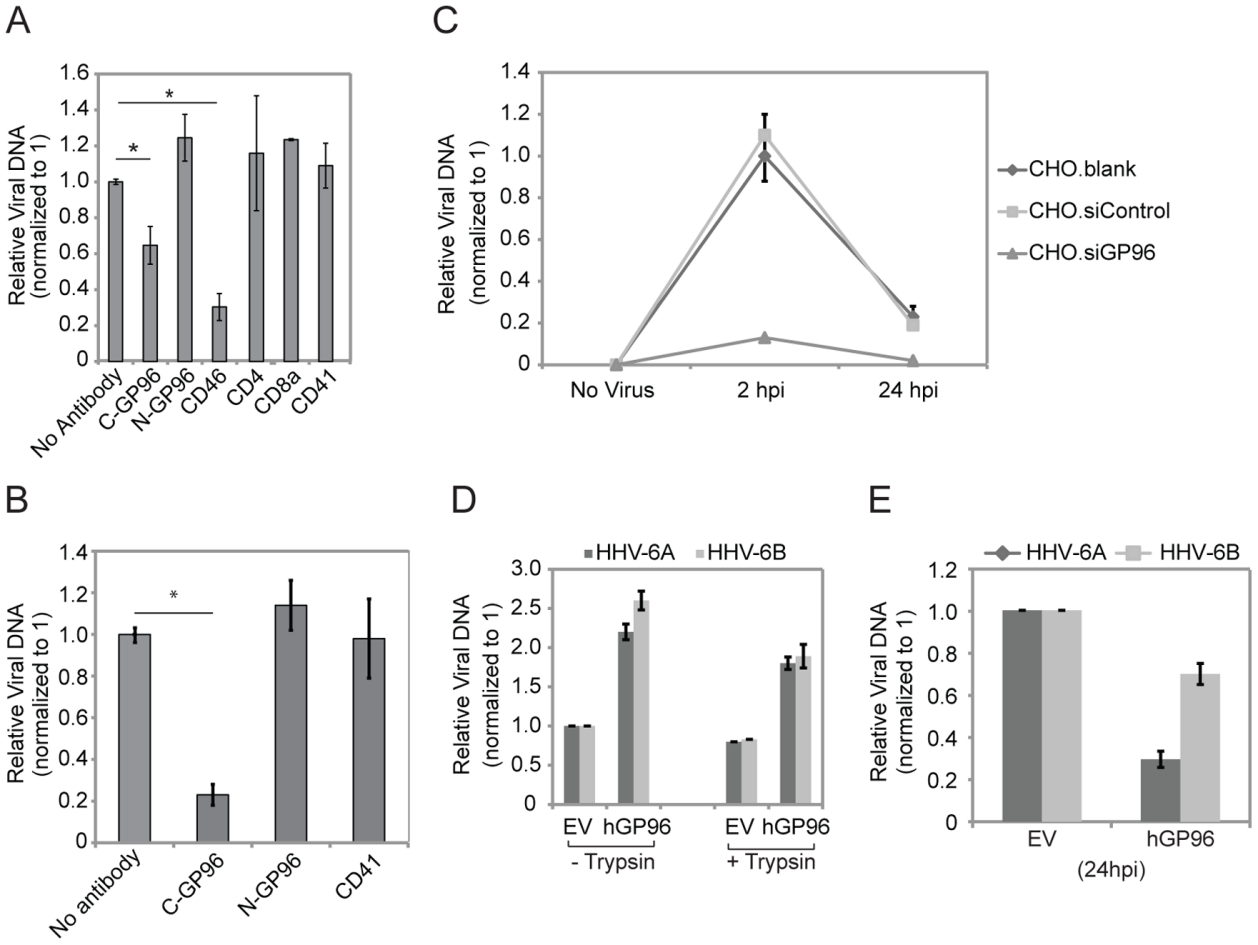
HHV-6 interacts with GP96. (**A**) Antibodies raised against the C-terminal end of GP96 prevent HHV-6A binding. HeLa cells were incubated with different antibodies for 30 min (used at 50 µg/ml) and then exposed to HHV-6 at the MOI of 1. After 1 h at 37°C, the cells were washed extensively and total DNA was extracted. The amount of viral particles bound to HeLa cells was measured by qPCR. Data represent the mean ± SEM of three independent experiments. *, p≤0.05. Statistical analysis was based on the Student t-test. (**B**) Similar experiments were performed in fresh PBMCs. Fresh PBMCs were isolated and stimulated with PHA for 2 days prior to the experiments. C-GP96, antibody raised against C-terminal of GP96; N-GP96, antibody raised against N-terminal of GP96. (**C**) Knock down of GP96 abolishes HHV-6 entry into CHO-K1 cells. CHO-K1 cells were transfected with siRNA against GP96 (siGP96). In parallel, cells were transfected with scrambled siRNAs (siControl) as a control. Blank transfected (CHO.blank) as well as siGP96 and siControl transfected cells were washed thoroughly and added with HHV-6A for 2 hrs after which cells were washed with trypsin for 3–5 minutes at 37°C followed by extensive washing with PBS to remove any unbound HHV-6 particles. Cells were then incubated for another 24 hrs in the absence of viral particle in the culture media and subsequently processed for quantification of viral DNA amount by qPCR. (**D**) Transient over-expression of human GP96 in CHO-K1 cells induces HHV-6 entry. CHO-K1 cells were transfected with a construct expressing human GP96 (hGp96). Empty vectors were transfected in parallel and were used as control. 48 h after transfection, cells were washed thoroughly and were infected with HHV-6A and -6B at an MOI of 10 for 2 h. A parallel set of infected cells was trypsinized to remove unbound viral DNA just before DNA extraction. Cells were washed extensively and total DNA was extracted. Viral DNA amount was quantified by qPCR. (**E**) Transient over-expression of human GP96 in CHO-K1 cells decreases HHV-6 survival. CHO-K1 cells were transfected with a construct expressing human GP96 (hGp96). Empty vectors were transfected in parallel and were used as control. 48 h after transfection, cells were washed thoroughly and were infected with HHV-6A and -6B at an MOI of 10 for 2 hrs and were processed as mentioned above. Viral DNA amount was quantified by qPCR.

To further validate a role of GP96 for HHV-6 entry, we used monoclonal antibodies against C- and N-terminus of GP96 to block binding of the virus to GP96. A monoclonal antibody against the C-terminus of GP96 (anti-GP96 C-term) decreased viral entry up to 50% (Figure 4A). In contrast, an anti-GP96 N-terminus antibody as well as a non-related anti-CD4, -CD8a, -CD41 antibodies could not decrease the number of HHV-6 entering into HeLa cells.

We performed similar experiments in phytohemagglutinin (PHA) stimulated human peripheral blood mononuclear cells (PBMCs) and found an even stronger down regulation of HHV-6 entry into PBMCs when the interaction of HHV-6 with GP96 was inhibited with anti-GP96 C-terminus (Figure 4B). Thus, these data support an association between the C-terminus of GP96 and HHV-6 viral particles.

We further tested the role of GP96 for viral entry into CHO-K1 cells, a unique system to study HHV-6 receptors since these cells express very low levels of GP96 (Figure S3) [27] in the absence of CD46 [18]. Due to the very weak binding of viral particles in CHO-K1 cells, short-term (30 min–1 h) binding experiments as performed in HeLa cells (Figure 2D) were not possible in these cells. We therefore focused on viral entry in these cells. Interestingly, HHV-6A entered CHO-K1 cells at low frequency and viral DNA was efficiently cleared within 24 h post infection (Figure 4C). siRNA-mediated suppression of the low levels of GP96 expression (Figure S3) completely prevented viral entry into CHO-K1 cells (Figure 4C). Transient over expression of human GP96 (hGP96) in CHO-K1 cells caused increased HHV-6A entry (Figure 4D) but also faster degradation of HHV-6A DNA (Figure 4E). To exclude that HHV-6A particles unspecifically stick to the cell surface during the incubation and to demonstrate entry of HHV-6 into CHO-K1 cells expressing hGP96, HHV-6A exposed cells were trypsinized and washed extensively before DNA extraction (Figure 4D). Quantification of viral DNA showed that the majority of viral particles had entered the cell during the incubation process. Thus, GP96 supports viral internalization in the absence of CD46 but also interferes with viral DNA survival and replication.

### Cell surface expression of GP96 is modulated during HHV-6 infection

**Figure 5:**
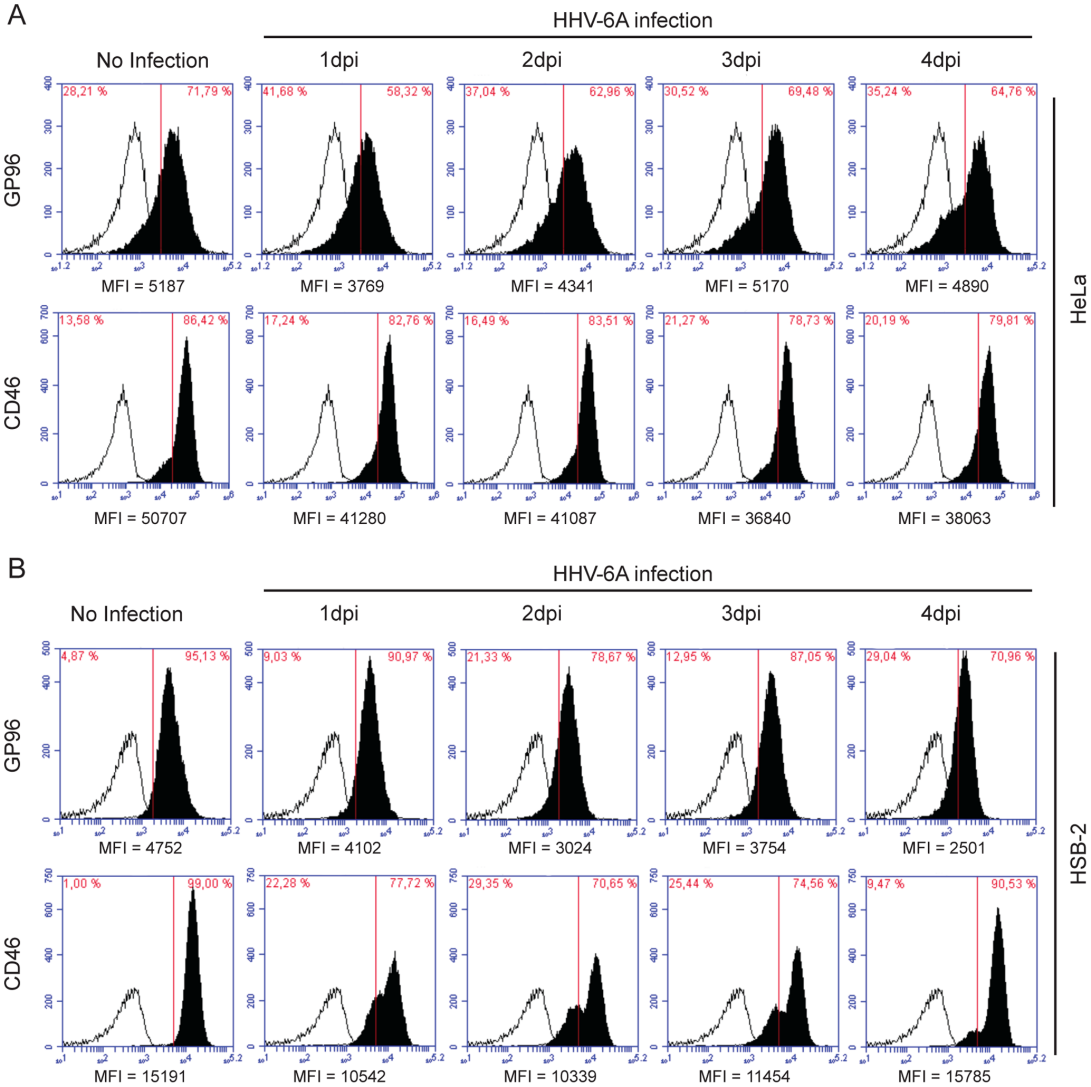
Cell surface expression of GP96 is modulated during HHV-6 infection. Cell surface expression dynamics of GP96 and CD46 in response to HHV-6A infection was determined in (**A**) HeLa and (**B**) HSB-2 cells. Cells were infected with HHV-6A for indicated time points and cell surface expression of CD46 and GP96 was performed by flow cytometry (solid profiles); background fluorescence levels were measured using an isotype specific antibody (empty profiles). Mean fluorescence intensity (MFI) for each analysis is indicated below the respective figure.

To further unravel the cell surface expression dynamics of GP96 and CD46 during HHV-6A infection, we studied their cell surface expression in HeLa and HSB-2 cells by flow cytometry. Under abortive viral infection in HeLa cells, we observed initial decrease in cell surface CD46 as well as GP96 expression (Figure 5A and Figure S4), which later returned back to basal level by 3–4 days post infection (dpi). In HSB-2 cells competent for productive HHV-6 infection, cell surface GP96 expression was continuously decreased until 4 dpi (Figure 5B and Figure S4). However, cell surface expression of CD46 was decreased in HSB-2 cells (Figure 5B) during initial 2–3 dpi, but returned to basal level by 4dpi. Similar experiments were carried out in fresh PBMCs without PHA stimulation. PHA stimulation is known to induce HHV-6 infection in PBMCs possibly because of dynamic changes in the cell surface expression pattern of many receptors as well as possible activation of multiple signaling pathways. To avoid this global effect, we preferred to use PBMCs without PHA stimulation, which is known to be less efficient for HHV-6 infection. As expected, cell surface GP96 expression was initially increased upon HHV-6A infection (Figure S4), which later decreased to some extent. However, CD46 expression was marginally down regulated during the first 2 days of viral infection (Figure S4). Together with the data on stability of viral DNA, the changes in cell surface expression of receptors may indicate that constant cell surface expression of GP96 possibly through a suitable recycling pathway support host cell defense against HHV-6 infection by directing the virus to a degradation pathway. In cells permissive for productive viral infection, absence of enough cell surface GP96 during later stages of infection, might allow a survival fate for HHV-6.

### GP96 allows HHV-6 entry in the absence of CD46

**Figure 6:**
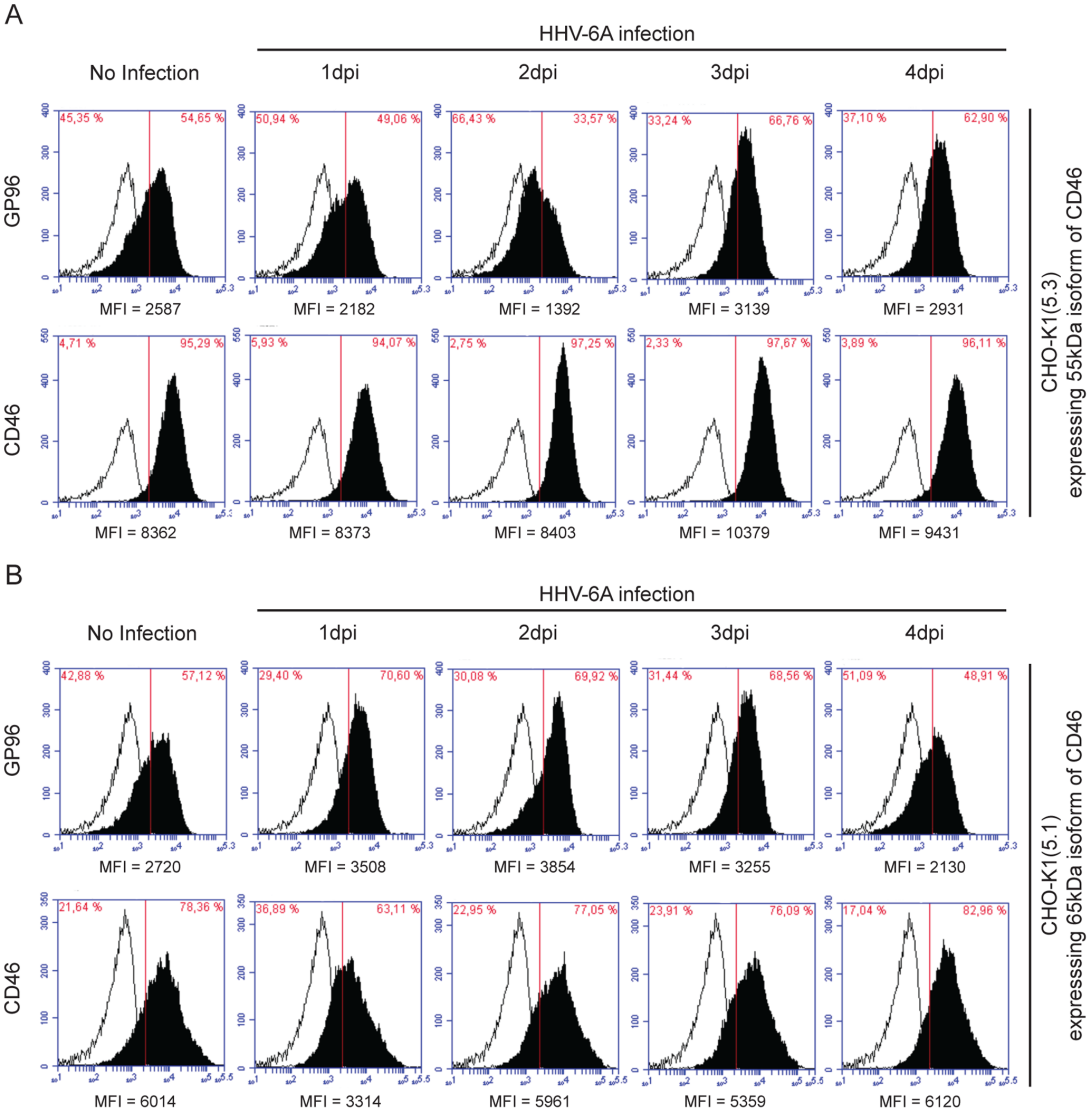
Association of CD46 with GP96 during HHV-6A infection. (**A**) Cell surface expression dynamics of GP96 in CHO-K1 cells stably expressing 55 kDa isoform of CD46. CHO-K1(5.3) cells expressing the 55 kDa isoform of CD46 were infected with HHV-6A for indicated time points. CD46 and GP96 cell surface expression were analyzed by flow cytometry (solid profiles); background fluorescence levels were measured using an isotype specific antibody (empty profiles). (**B**) Cell surface expression dynamics of GP96 in CHO-K1 cells stably expressing 65 kDa isoform of CD46. CHO-K1(5.1) cells expressing the 65 kDa isoform of CD46 were infected with HHV-6A for indicated time points. CD46 and GP96 cell surface expression were analyzed by flow cytometry (solid profiles); background fluorescence levels were measured using an isotype specific antibody (empty profiles). Mean fluorescence intensity (MFI) for each analysis is indicated below the respective figure.

Cells express two major CD46 isoforms of 65 kDa and 55 kDa. To define the role of these isoforms for HHV-6A binding in the presence of GP96, two additional previously well characterized CHO-K1 derived cell lines expressing either the 65 kDa or 55 kDa isoform of CD46 were employed [28]. In the cell line expressing the 55 kDA CD46 isoform, cell surface expression of GP96 decreased during the first 2 days of HHV-6A infection whereas cell surface CD46 (55 kDa) remained stable (Figure 6A and Figure S5). In contrast, infection of the cell line with the 65 kDA CD46 isoform resulted in an initial increase of surface GP96 (Figure 6B), which started to decrease 1day post infection. But we observed down-regulation of cell surface CD46 expression in these cells during the first 3 days of virus infection (Figure 6B and Figure S5). Thus, our results suggests possible binding and uptake of HHV-6A in CHO-K1 cells through GP96 in the absence of the 65 kDa isoform of CD46, whereas the major viral uptake was mediated through the 65 kDa isoform of CD46.

## Discussion

CD46 belongs to the regulators of complement activation (RCA) family, which prevents spontaneous activation of complement on autologous cells. It is widely accepted as a host receptor for HHV-6A [1], [29]. HHV-6A glycoprotein-H, -L and -Q complex associates with human CD46 [4], [30]. In addition, several lines of evidence support an important role of CD46 in HHV-6A infection. Human hematopoietic progenitor cells express CD46 on their surface, and hence are susceptible to HHV-6A infection [31]. However, there is also evidence that other human HHV-6A receptors than CD46 exist. CD46 is expressed in almost all human nucleated cells, but not all of the human nucleated cells are permissive for HHV-6 infection. Further, CD46 acts as a receptor for measles virus [32] and CD46 transgenic mice are susceptible to certain strains of measles virus (MV) [33], [34] but are not permissive to HHV-6 infection. Also, HHV-6A, but not HHV-6B, recognizes CD46 reflecting difference in host cell tropism of these virus subtypes. Thus, these findings argue for a broader range of human proteins that might influence HHV-6 entry.

We followed an unbiased co-immunoprecipitation approach and detected 11 different host cell proteins, which specifically interacted with HHV-6 and/or viral glycoproteins. We observed direct interaction of heat shock chaperone protein family member GP96 with HHV-6. When GP96 was silenced by siRNAs or shRNAs, we observed a decrease in viral binding and subsequent entry in both HeLa and HSB-2 cells. We followed some of the classical approaches to study viral receptors. By using monoclonal antibodies against GP96 we demonstrated that the C-terminal end of GP96 possibly interacts with HHV-6 and facilitates the internalization of viral particles. These results do not disproof the binding of a N-terminal peptide binding sites in GP96, which have been demonstrated before [35]. Although silencing of GP96 expression decreased the viral binding, it was not completely prevented. Similarly, monoclonal antibodies directed against the C-terminal end of GP96 could only partially decrease the viral entry. These results suggest the involvement of multiple receptors for viral attachment and entry.

A potential viral receptor can either be ubiquitously expressed on the host cell surface (like CD46) or can be transported to the surface immediately upon virus infection. One such example for the later type of receptor is non-muscle myosin IIA, which acts as a functional entry receptor for herpes simplex virus-1 [36] and is up-regulated on the host cell surface immediately after viral infection. Cell surface expression of HIV-1 receptor CCR5 is dependent on the rate of receptor internalization and recycling [37]. As one would expect from a receptor for HHV-6, our results showed constant changes in cell surface exposure of GP96 at early infection time points, but few days post infection returned to basal levels. Thus our results support a role of GP96 in HHV-6 binding and entry. Interestingly, in the absence of GP96, the amount of virus DNA increased 2-fold within 24 h of infection in both, cells that either do not allow productive infection (HeLa) or are productively infected (HSB-2). We also show that cells lacking GP96 allow better expression of viral early proteins thus supporting better viral replication. In HeLa cells, HHV-6 virus particles are sequentially degraded thereby possibly clearing the viral infection within few days. The increase in viral DNA in HeLa cells with suppressed GP96 expression suggests that GP96 is involved in the initiation of innate immunity that facilitates viral degradation. GP96 cross-present antigens and can be loaded with synthetic peptides *in vitro* that can elicit antigen-specific responses *in vivo* [35], [38], [39]. In addition, it has been suggested that such cross-presentation is based on the transfer of proteasome substrates (whole proteins, or large fragments of such), rather than peptides [40]. Our data supports this function of GP96. But the dependence of the productive viral life cycle on GP96 argues for additional deciding factors, which helps the virus to overcome the cellular autoimmunity pathway inside HSB-2 cells. GP96 is an ER associated protein and shows a retrograde transport to cell surface in many *in vivo* and *in vitro* conditions. Availability of GP96 on cell surface can be crucial factor for the host cell specificity of HHV-6 but its fate inside the host cell is dependent on further association of GP96 with other proteins. CD91 [41] and Toll-like receptors bind to GP96 and are responsible for the downstream signaling in antigen presenting cells (APC) [42]. Cells deficient in GP96 lack functional TLR [43] and thus lack efficient innate immune responses against a wide variety of pathogens. A general down-regulation of innate immunity in GP96-depleted cells may thus explain the increased level of HHV-6 DNA despite reduced initial infection.

It is very likely that additional proteins besides CD46 and GP96 are involved in HHV-6 infection. During *Neisseria gonorrhoeae* infection, bacterial pili initiate signaling pathways via CD46 [44] that recruit cortical plaques directly to the adherent bacteria [45]. The PorB porin attaches to heat shock protein GP96 and the scavenger receptor SREC [15]. All these interaction facilitate infection of *N. gonorrhoeae* with the human cell. Possibly HHV-6 utilizes a similar approach for its entry into the host cell and therefore CD46 is very likely not the only receptor protein for HHV-6 binding. A complex of proteins involving GP96 and CD46 rather may provide an explanation for the selectivity of HHV-6 for entry into host cells.

Binding of HHV-6 to its receptor might not be the only criteria for viral entry. The internalization of HHV-6 is also crucial and should be facilitated by its binding to certain ligands on cell surface. Not much work has been done in this regard so far. Measles hemagglutinin binding to CD46 down regulates the cell surface expression of CD46 [46]. Therefore, internalization of CD46 from the cell surface is thought to be important for functioning of CD46. CD46 is constitutively internalized via clathrin-coated pits, traffics to multivesicular bodies, and is recycled to the cell surface. However, cross-linking of CD46 at the cell surface induces macropinocytosis of CD46, and leads to the degradation of cell surface CD46 [47]. We identified clathrin heavy chain isoform 1 and 2 (CHC22 and CHC17) co-precipitating with HHV-6A and 6B (Table 1) thus indicating a possibility of internalization and transport of HHV-6 via clathrin coated pits and vesicles. We could not co-immunoprecipitate CD46 or CD136 in our virus co-immunoprecipitation experiments (Table 1). This may also be due to the fact that we sequenced only clearly defined protein bands, which were not observed in control samples. A wider approach should be taken to identify more candidate proteins that directly interact with HHV-6.

We detected increased amounts of viral DNA in the absence of GP96. This indicates a complex association of GP96 with cell surface receptors, which together determine the fate of the virus inside the host cell (Figure S6). In the absence of GP96, HHV-6 might bind to CD46 directly, which decreases its chance of entering into the cell. But, it might push the virus into a different mode of endocytosis (possibly micropinocytosis), which protects the virus from cellular immunity and allows its survival. Our results suggest that productive viral infection is dependent on a delicate balance between cell surface GP96 expression and several other receptors including CD46.

## Supporting Information

Figure S1 **Identification of HHV-6 interacting proteins by co-immunoprecipitation.** Protein complexes interacting with HHV-6 envelope glycoproteins were isolated by co-immunoprecipitation, separated by SDS-PAGE and visualized by silver staining. Molecular weight markers (MW marker) are indicated on the right, arrowheads point to proteins identified by mass spectrometry. Wpc, with pre-clearing of HeLa protein lysate.
(TIF)

Figure S2 **Validation of GP96 down regulation.** (A) GP96 expression was analyzed by Western blotting in HeLa and two different single cells clones (K1 and K2) of HeLa with stable knock down of GP96 (HeLa.shGP96). (B) Immunoblot showing decreased expression of GP96 in presence of siRNA against human GP96. siRNA against lucifearse gene (siLUC) was used as control. (C) GP96 expression in HeLa cells with stable knock down of GP96. GP96 expression was knocked down in HeLa cells (HeLa.shGP96) using lentivirus-mediated shRNA. Mock lentivirus backbone served as a control (HeLa.mock). Human GP96 expression was rescued using transient expression of dsRed-tagged human GP96. Actin served as loading control. (D) HHV-6A early protein p41 expression is induced in the absence of GP96. HHV-6 p41 expression was studied by confocal laser microscopy in HeLa and HeLa.shGP96 cells after 96 hrs of HHV-6A infection. The scale bar represents 10 μ.
(TIF)

Figure S3 **GP96 supports HHV-6 entry in absence of CD46.** (A) CHO-K1 cells express low amounts of GP96, which is upregulated after HHV-6A and -6B infection. Immunoblot showing GP96 expression in CHO-K1 cells before and after HHV-6 infection. (B) Silencing GP96 in CHO-K1 cells. CHO-K1 cells were transfected with siRNA against GP96 (siGP96) and the efficiency of GP96 silencing was assayed by immunoblotting. Scrambled siRNAs (siControl) were used as a control.
(TIF)

Figure S4 **Cell surface expression pattern of CD46 and GP96 during HHV-6A infection in HeLa and HSB-2 cells.** (A) Cell surface expression dynamics of GP96 and CD46 in HeLa cells. HeLa cells were infected with HHV-6A for indicated time points. CD46 and GP96 cell surface expression were analyzed by flow cytometry without cell permeabilization. Mean fluorescence intensity (MFI) values are plotted as line graphs. (B) Similar experiment was carried out in HSB-2 cells. Mean fluorescence intensity (MFI) values are plotted as line graphs. Data represents mean MFI values of three independent experiments.
(TIF)

Figure S5 **Association of different isoforms of CD46 with GP96 during HHV-6A infection.** (A) Cell surface expression dynamics of GP96 in CHO-K1 cells stably expressing 55 kDa isoform of CD46. CHO-K1(5.3) cells expressing the 55 kDa isoform of CD46 were infected with HHV-6A for indicated time points. CD46 and GP96 cell surface expression were analyzed by flow cytometry. Mean fluorescence intensity (MFI) values are plotted as line graphs. (B) Cell surface expression dynamics of GP96 in CHO-K1 cells stably expressing 65 kDa isoform of CD46. CHO-K1(5.1) cells expressing the 65 kDa isoform of CD46 were infected with HHV-6A for indicated time points. CD46 and GP96 cell surface expression were analyzed by flow cytometry. Mean fluorescence intensity (MFI) values are plotted as line graphs. Data represents mean MFI values of three independent experiments.
(TIF)

Figure S6 **Graphical abstract showing the possible role of GP96 and CD46 during HHV-6 infection.** PM, plasma membrane; ER, endoplasmic reticulum.
(TIF)
