# Supplementary figures and images for: GP96 Interacts with HHV-6 during Viral Entry and Directs It for Cellular Degradation
Source: PLoS One. 2014 Dec 3;9(12):e113962. doi: 10.1371/journal.pone.0113962 (PMC4254946; doi:10.1371/journal.pone.0113962)

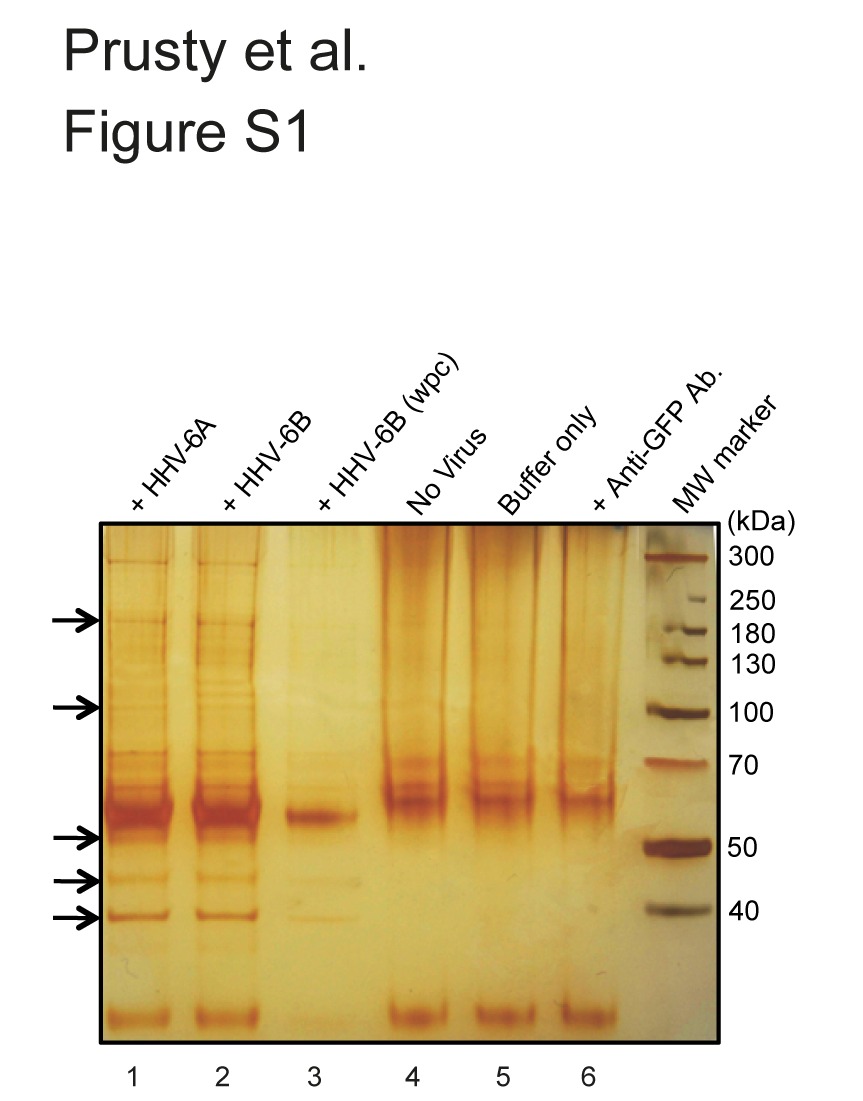

Supplement: Figure S1 — Identification of HHV-6 interacting proteins by co-immunoprecipitation. Protein complexes interacting with HHV-6 envelope glycoproteins were isolated by co-immunoprecipitation, separated by SDS-PAGE and visualized by silver staining. Molecular weight markers (MW marker) are indicated on the right, arrowheads point to proteins identified by mass spectrometry. Wpc, with pre-clearing of HeLa protein lysate. (TIF) [file pone.0113962.s001.tif]

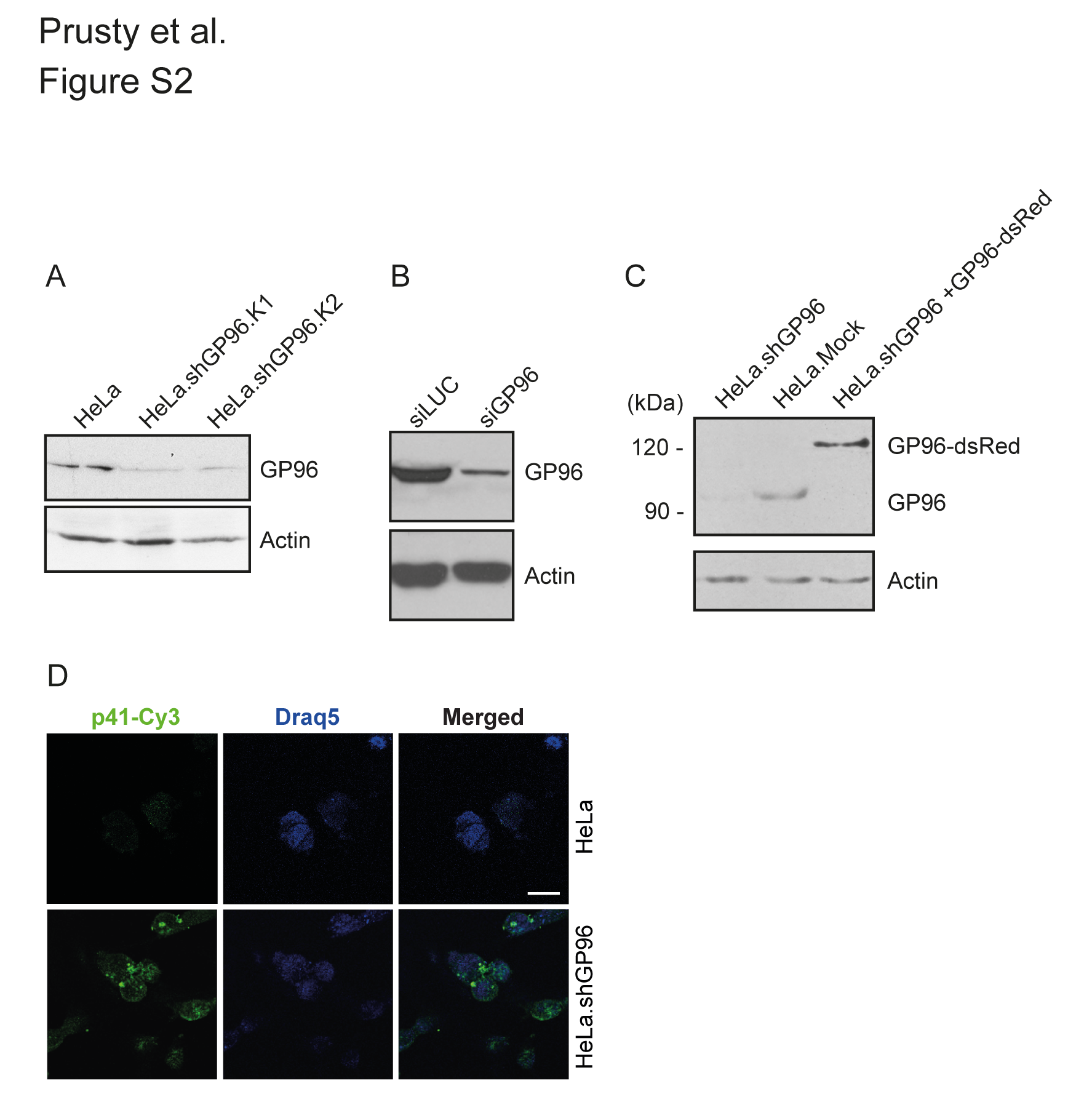

Supplement: Figure S2 — Validation of GP96 down regulation. (A) GP96 expression was analyzed by Western blotting in HeLa and two different single cells clones (K1 and K2) of HeLa with stable knock down of GP96 (HeLa.shGP96). (B) Immunoblot showing decreased expression of GP96 in presence of siRNA against human GP96. siRNA against lucifearse gene (siLUC) was used as control. (C) GP96 expression in HeLa cells with stable knock down of GP96. GP96 expression was knocked down in HeLa cells (HeLa.shGP96) using lentivirus-mediated shRNA. Mock lentivirus backbone served as a control (HeLa.mock). Human GP96 expression was rescued using transient expression of dsRed-tagged human GP96. Actin served as loading control. (D) HHV-6A early protein p41 expression is induced in the absence of GP96. HHV-6 p41 expression was studied by confocal laser microscopy in HeLa and HeLa.shGP96 cells after 96 hrs of HHV-6A infection. The scale bar represents 10 μ. (TIF) [file pone.0113962.s002.tif]

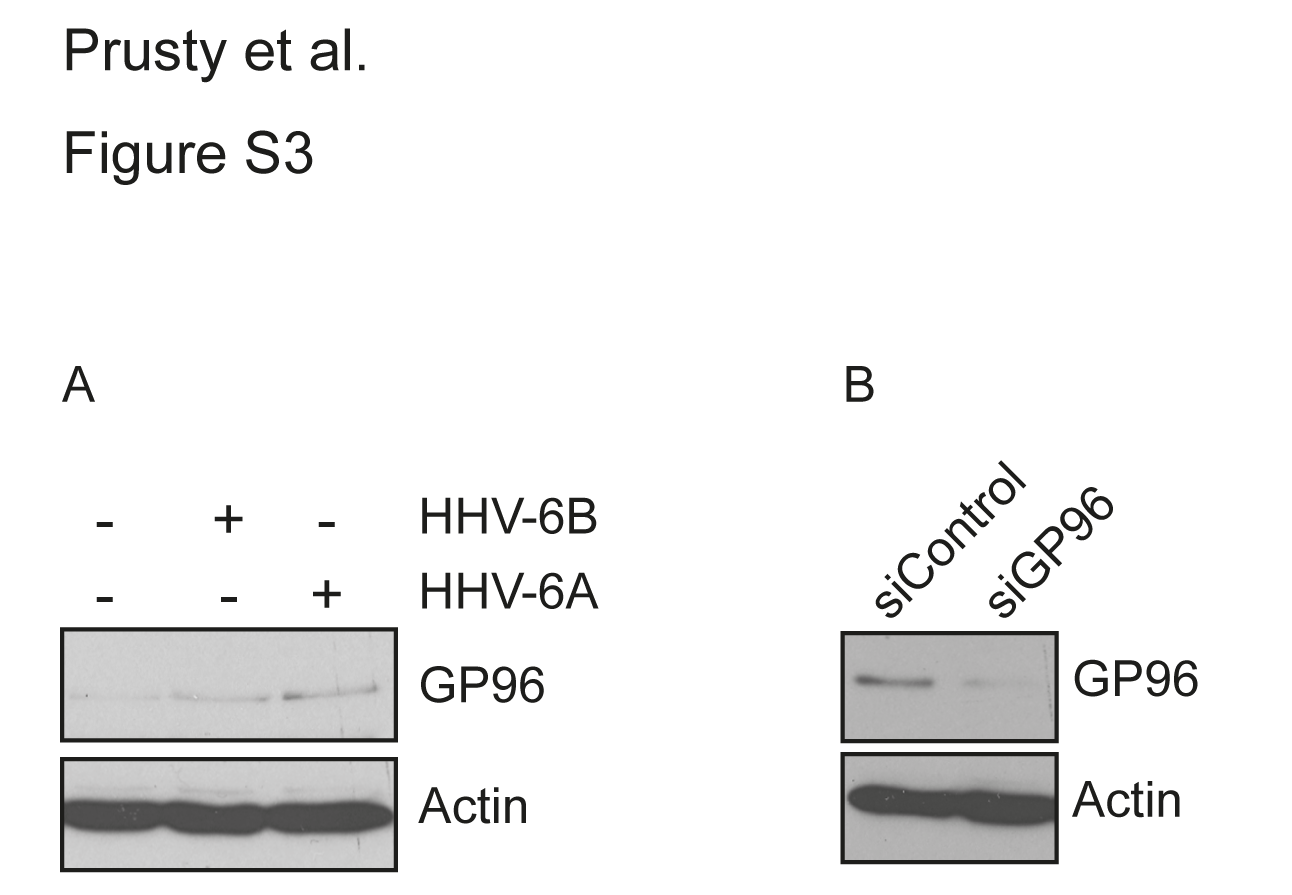

Supplement: Figure S3 — GP96 supports HHV-6 entry in absence of CD46. (A) CHO-K1 cells express low amounts of GP96, which is upregulated after HHV-6A and -6B infection. Immunoblot showing GP96 expression in CHO-K1 cells before and after HHV-6 infection. (B) Silencing GP96 in CHO-K1 cells. CHO-K1 cells were transfected with siRNA against GP96 (siGP96) and the efficiency of GP96 silencing was assayed by immunoblotting. Scrambled siRNAs (siControl) were used as a control. (TIF) [file pone.0113962.s003.tif]

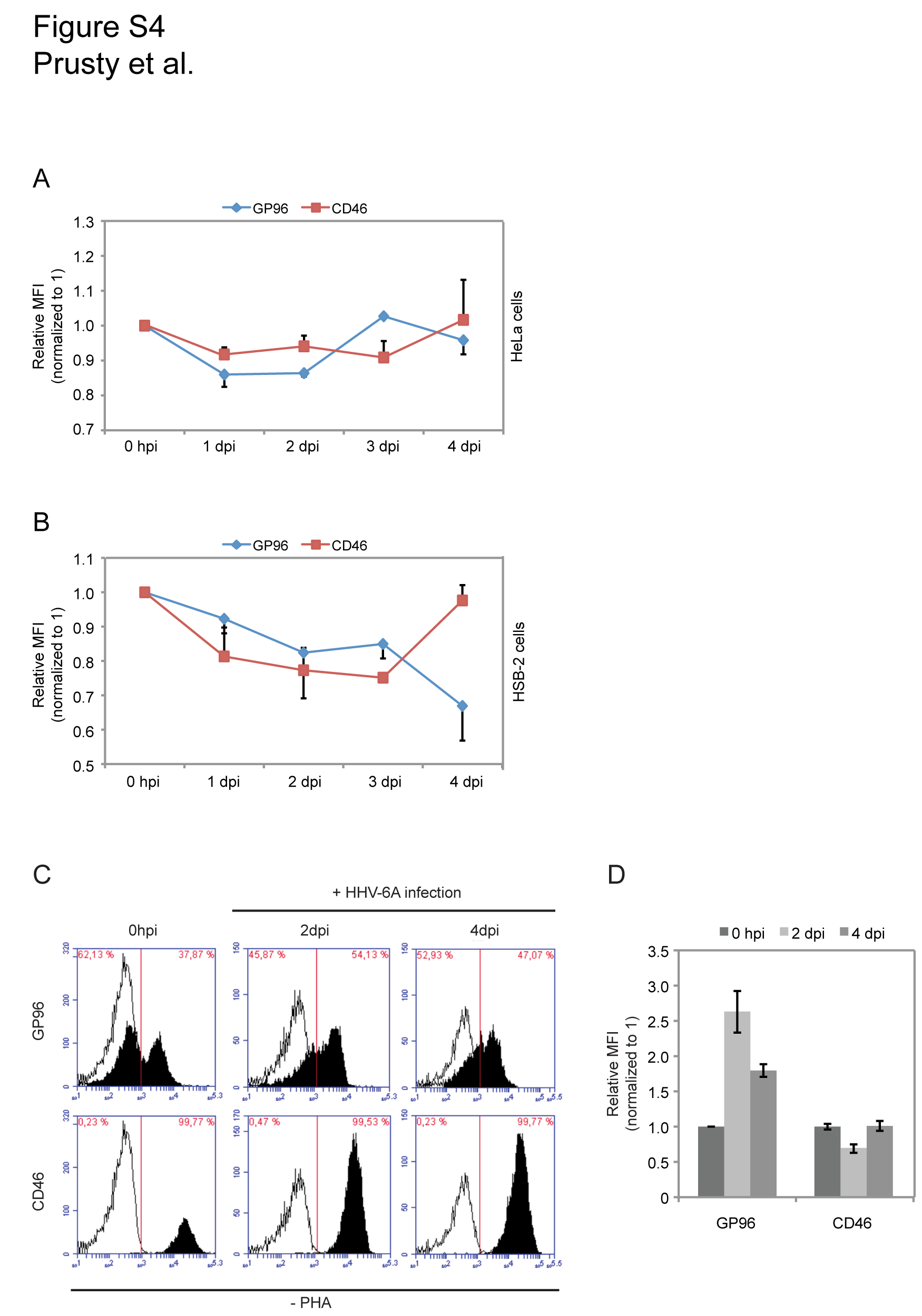

Supplement: Figure S4 — Cell surface expression pattern of CD46 and GP96 during HHV-6A infection in HeLa and HSB-2 cells. (A) Cell surface expression dynamics of GP96 and CD46 in HeLa cells. HeLa cells were infected with HHV-6A for indicated time points. CD46 and GP96 cell surface expression were analyzed by flow cytometry without cell permeabilization. Mean fluorescence intensity (MFI) values are plotted as line graphs. (B) Similar experiment was carried out in HSB-2 cells. Mean fluorescence intensity (MFI) values are plotted as line graphs. Data represents mean MFI values of three independent experiments. (TIF) [file pone.0113962.s004.tif]

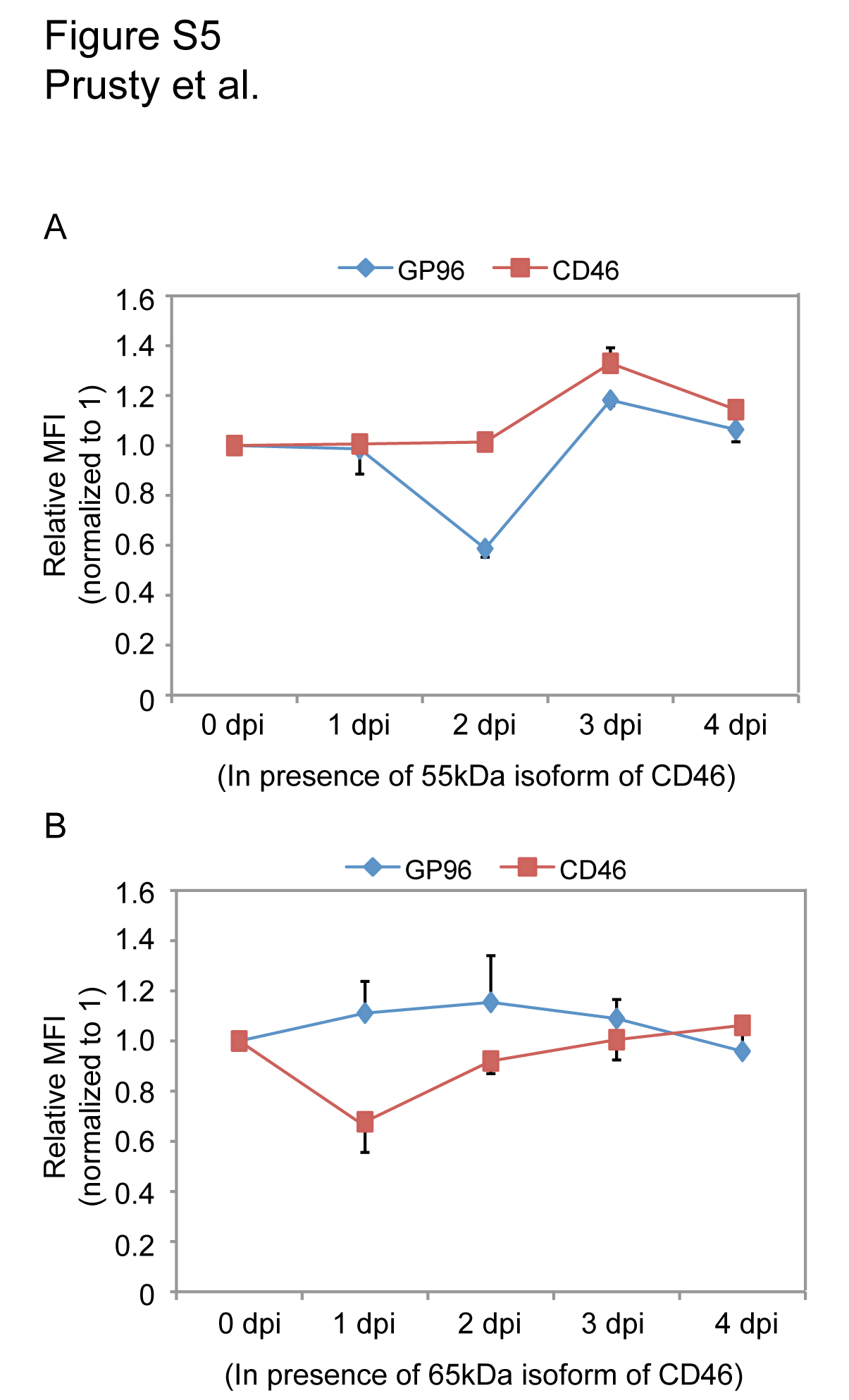

Supplement: Figure S5 — Association of different isoforms of CD46 with GP96 during HHV-6A infection. (A) Cell surface expression dynamics of GP96 in CHO-K1 cells stably expressing 55 kDa isoform of CD46. CHO-K1(5.3) cells expressing the 55 kDa isoform of CD46 were infected with HHV-6A for indicated time points. CD46 and GP96 cell surface expression were analyzed by flow cytometry. Mean fluorescence intensity (MFI) values are plotted as line graphs. (B) Cell surface expression dynamics of GP96 in CHO-K1 cells stably expressing 65 kDa isoform of CD46. CHO-K1(5.1) cells expressing the 65 kDa isoform of CD46 were infected with HHV-6A for indicated time points. CD46 and GP96 cell surface expression were analyzed by flow cytometry. Mean fluorescence intensity (MFI) values are plotted as line graphs. Data represents mean MFI values of three independent experiments. (TIF) [file pone.0113962.s005.tif]

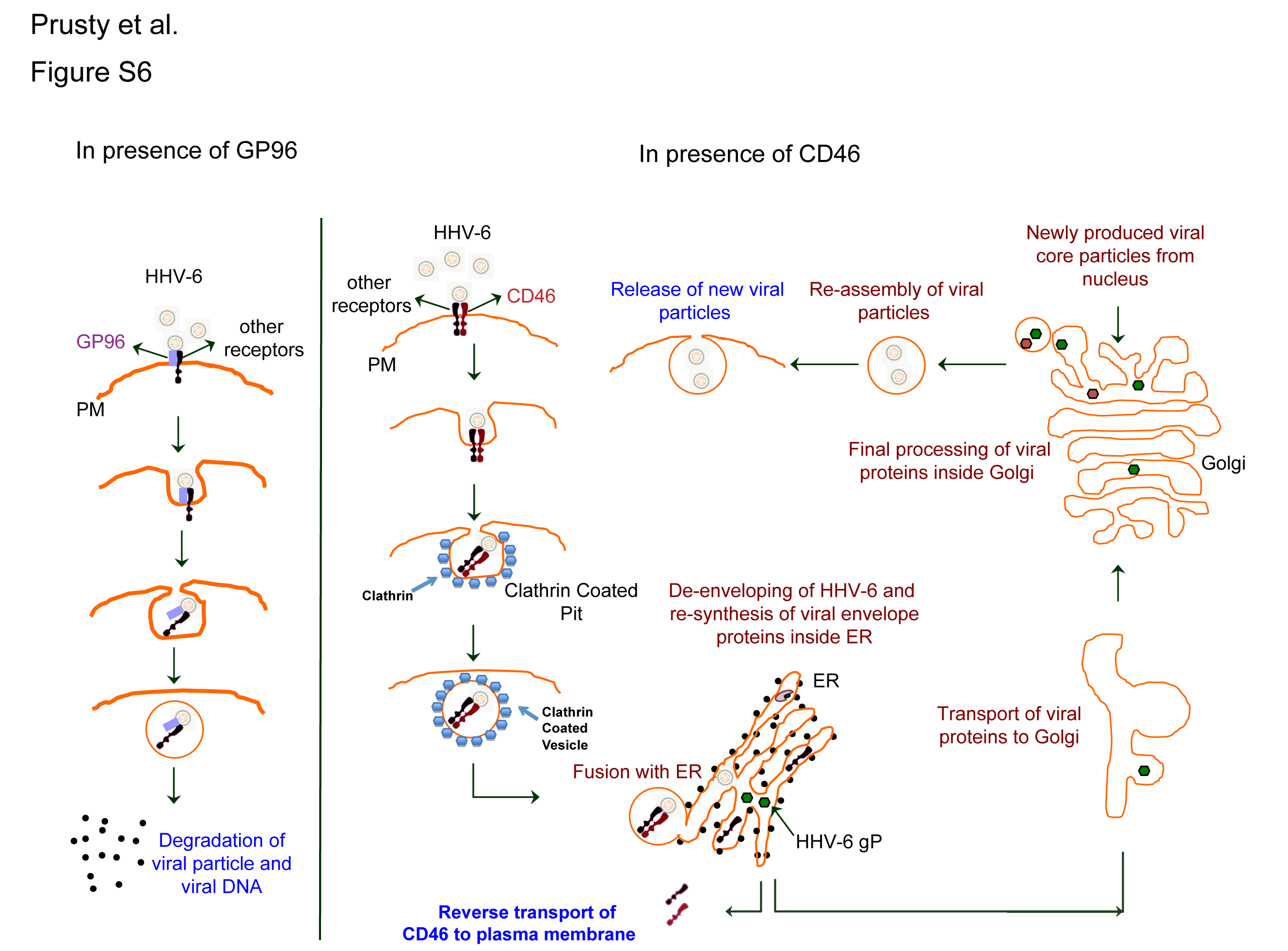

Supplement: Figure S6 — Graphical abstract showing the possible role of GP96 and CD46 during HHV-6 infection. PM, plasma membrane; ER, endoplasmic reticulum. (TIF) [file pone.0113962.s006.tif]
